# 53-year-old Woman with Opsoclonus-Myoclonus Syndrome

**DOI:** 10.5811/cpcem.53141

**Published:** 2026-04-14

**Authors:** Taylor Stephens, Bryan Imhoff, Janak Patel

**Affiliations:** * The University of Kansas School of Medicine, Kansas City, Kansas; † The University of Kansas Health System, Department of Emergency Medicine, Kansas City, Kansas

**Keywords:** opsoclonus, myoclonus, eye movements, case report

## Abstract

**Case Presentation:**

We present the case of a 53-year-old female with darting eye movements and difficulty walking who was found to have opsoclonus-myoclonus syndrome only after multiple presentations to emergency departments over five days.

**Discussion:**

Adult-onset opsoclonus-myoclonus syndrome is a rare central nervous system disease typically associated with paraneoplastic or idiopathic etiologies. With non-specific symptom presentation, this condition is commonly misdiagnosed in adults, leading to diagnostic delays and long-term motor and cognitive sequelae.

## CASE PRESENTATION

A 53-year-old woman with a history of migraine headaches presented to her third emergency department (ED) in five days for suspected nystagmus and difficulty walking. She presented to the first ED reporting that her vision had “trouble focusing” and was documented to have eyes “darting back and forth when talking.” No acute findings on computed tomography head without contrast and magnetic resonance imaging head with and without contrast prompted discharge with a diagnosis of nystagmus with instructions to follow up with her ophthalmologist. Her ophthalmologist found no primary vision problems and referred her to otolaryngology (ENT) for “vestibular concerns.” The ENT office indicated she would be better served by a neurologist. Without improvement in her symptoms, she presented to a second ED two days after her primary encounter. There, her physical exam noted “visible horizontal nystagmus” and an unstable gait. Given previous negative imaging and established neurology follow-up, she was discharged. Two days later, she presented to our ED with worsening vision and gait that required significant familial assistance. On physical exam, she demonstrated persistent bilateral opsoclonus (video), significant left lower extremity myoclonus, and ataxia.

Neurology was consulted in our ED, and she was admitted to their service for further workup. While admitted, she received additional advanced imaging, lumbar puncture, and paraneoplastic and autoimmune panels with unremarkable results. Neurology diagnosed the patient with idiopathic opsoclonus-myoclonus syndrome and initiated treatment with intravenous (IV) methylprednisolone and IV immunoglobulin (IG) infusions followed by an oral prednisone taper at discharge, along with monthly IVIG and steroid infusions for 12 months. After three months of treatment, the patient reported feeling 80% back to baseline. Her myoclonus and ataxia had resolved, and opsoclonus had improved. Her cerebellar symptoms, including end point tremor, had improved with the addition of propranolol, providing a secondary anxiolytic benefit.

## DISCUSSION

Opsoclonus-myoclonus syndrome is a central nervous system disease primarily affecting toddlers and is often associated with underlying malignancy.^1^ Adult onset is rare (< 0.2 cases per million) and typically associated with paraneoplastic or idiopathic (commonly para-infectious) etiologies.^2^,^3^ Our patient’s abnormal, erratic eye movements prompted a wider differential diagnosis to include opsoclonus-myoclonus syndrome as referenced in the original ED history and physical. With non-specific symptom presentation, this condition is commonly misdiagnosed in the adult population, leading to problematic diagnostic delays averaging 11 weeks following symptom onset.^3^

A common misdiagnosis is nystagmus, which consists of horizontal rhythmic and slow oscillation, whereas opsoclonus involves horizontal, vertical, and torsional saccades.^1^ This delay in diagnosis can result in long-term motor and cognitive deficits, commonly ataxia and residual dysarthria along with possible progression of underlying small-cell lung cancer, breast cancer, and ovarian cancer in paraneoplastic cases.^1^,^2^ With suspicion for opsoclonus-myoclonus syndrome, physicians should engage an integrated team of specialists including neurology, oncology, and immunology. Moreover, involvement of occupational and mental health therapists may improve prognosis and residual deficits.^4^

To initiate the process of healthcare team collaboration, emergency physicians should have clinical suspicion for opsoclonus when abnormal eye movements appear more erratic, rapid, and/or bidirectional than nystagmus, suggesting a pathologic presentation. Diagnosis can be difficult and is purely based on physical exam findings, given that the patient’s imaging and lab tests are typically negative. Nonetheless, prompt initiation of treatment is necessary to minimize the risk of long-term motor and cognitive sequelae.^1^

*CPC-EM Capsule*

What do we already know about this clinical entity?

*While opsoclonus-myoclonus syndrome most commonly affects children, it should remain on the differential for adults to avoid delay in diagnosis and treatment*.

What is the major impact of the image(s)?

*Video shows the characteristic non-directional eye movements in opsoclonus-myoclonus syndrome as opposed to nystagmus, a common misdiagnosis*.

How might this improve emergency medicine practice?

*Understanding the nuances of eye movement disorders prevents diagnostic delays and progression of symptoms to functional impairment*.

## Supplementary Information

Video Finding of bilateral opsoclonus on physical exam of 52-year-old woman who was diagnosed only after presentation to three emergency departments.
